# ALZHEIMER'S DISEASE AND RELATED DEMENTIAS RISK AND PSYCHOSOCIAL HEALTH NEEDS OF OLDER ADULT IMMIGRANTS AND REFUGEES

**DOI:** 10.1093/geroni/igac059.870

**Published:** 2022-12-20

**Authors:** Hafifa Siddiq, Nahla Kayali, Kristine Ajrouch

**Affiliations:** Charles R. Drew University, Garden Grove, California, United States; Access California Services, Anaheim, California, United States; Eastern Michigan University, Ypsilnati, Michigan, United States

## Abstract

To establish priorities for an intervention addressing Alzheimer’s Disease and Related Dementias (ADRD) risk among older adult immigrants and refugees from Afghanistan and the Middle-East and North Africa (MENA). A multi-methods Community-Based Collaborative Action Research design was employed to gather qualitative and quantitative data. Twenty community leaders and stakeholders participated in interviews and community needs survey responses of 124 ethnically diverse adults over age 55 were examined. We analyzed community needs survey data using descriptive statistics. A step-wise Thematic Analysis was used to analyze interview transcripts. A major theme and standout priority was identified: needing culturally-sensitive services and resources addressing social isolation among the elderly. Other psychosocial priorities included: accessibility needs, caregiving support, safety and end-of-life resources, digital literacy, and English language services. This study exemplifies a priority-setting partnership between clinician-researchers and refugee service providers. The priorities highlighted ADRD risk factors and guide the collaborative development of a community-based intervention.

